# We are not even allowed to call them patients anymore: Conceptions about person‐centred care

**DOI:** 10.1111/hex.13887

**Published:** 2023-10-18

**Authors:** Sylvia Määttä, Ida Björkman

**Affiliations:** ^1^ Institute of Health and Care Sciences, Sahlgrenska Academy University of Gothenburg Gothenburg Sweden; ^2^ University of Gothenburg Centre for Person‐Centred Care (GPCC), Sahlgrenska Academy University of Gothenburg Gothenburg Sweden

**Keywords:** conceptions, implementation, person‐centred care

## Abstract

Although the implementation of person‐centred care (PCC) has gained momentum in healthcare systems, it is not always obvious why it is an important objective in clinical settings and educational institutions that train future healthcare professionals. Various conceptions about PCC have flourished, such as that it only works in certain healthcare contexts, it is the same as the patient making all their own healthcare decisions, it is too demanding for most patients, the word ‘patient’ may no longer be used, the care provided is already person‐centred, and that there is simply not enough time and resources to achieve it. This viewpoint paper aims to problematize these conceptions and may be used for didactical and reflective exercises that support the transition towards more person‐centred healthcare.

**Patient or Public Contribution:**

The Person Council for patients and carers at the University of Gothenburg provided focus group input on and validated the occurrence of the conceptions discussed in the present paper. The person council consists of a group of persons with many years of personal experiences of health care, either as patients and/or relatives/carers. One member of the person council who is also a designer and artist created the illustration for the article.

## 1 INTRODUCTION

National and international steering documents advocate for a shift towards person‐centred care (PCC).^1^, ^2^ Many scholars claim that it is problematic to give a precise definition of PCC as different definitions and understandings of PCC exist^3^, ^4^ For example, concepts like patient‐centred care, relationship‐centred care are similar and related but not interchangeble. Håkansson Eklund et al.^5^ describe that PCC builds on and develops patient‐centred care. However, the ideas of PCC generally describe healthcare practices that see and treat the patient as a person, characterized by mutual respect and co‐creation of health and care where the patient's knowledge, values, beliefs and preferences are considered.^6^ The patient is considered an active partner in and not a passive recipient of care. Our understanding of PCC is grounded on the components described by the Gothenburg Centre for Person‐Centred Care:

Person‐centredness is an ethical standpoint that guides our practical actions as fellow human beings and professionals. Person‐centred care entails a partnership between patient, their relatives, and professionals, in health and elderly care and rehabilitation. Based on carefully and perceptively listening to the narrative of the patient (often combined with the narratives of their relatives) and other examinations, a health plan is co‐created, containing goals and strategies for implementation, along with short and long‐term follow‐up.^7^

However, PCC is not always easy to achieve. Various conceptions have been seen in studies of higher education settings that train future healthcare professionals. This viewpoint article aims to discuss and problematize commonly held conceptions about PCC and explore how they may contribute to uncertainty about what it is, how it should be carried out, and its value. The conceptions were identified based on various sources. One of these sources was published literature on the implementation of PCC. In an interview study, a number of these conceptions were voiced at higher educational settings that train future healthcare professionals.^8^ In clinical settings, Fridberg et al.^3^ found that healthcare professionals perceived PCC as complicated, and although many were positive towards it, it was far from obvious to all professionals why or how it should be carried out. Another source consisted of findings from a focus group involving the Person Council for patients and caregivers at the Gothenburg University Centre for PCC. This council comprises individuals with extensive personal experiences within the healthcare system, either as patients, relatives, caregivers or a combination of these roles. During the focus group discussions, the council deliberated on their beliefs about PCC and contemplated the obstacles and drivers for implementing PCC within healthcare. Throughout the focus group session, the council members recognized the conceptions they had encountered in various contexts. Additionally, sources included our own experiences related to teaching PCC to students and the challenges in implementing PCC within healthcare settings. While there might be other conceptions regarding what PCC entails and what it does not, we believe that the ensuing conceptions are commonly held and notably significant. It's crucial to reflect upon these conceptions as they could potentially hinder the implementation of PCC:

1. PCC only works in certain healthcare settings.
2. PCC is the same as patients deciding on all aspects of their healthcare.
3. PCC is too demanding for most patients; they want healthcare professionals to make decisions.
4. We are no longer allowed to use the term ‘patients’.
5. We do not need to implement PCC because we already work in that manner.
6. We have neither the time nor the resources for PCC.

## 2 PCC ONLY WORKS IN CERTAIN HEALTHCARE SETTINGS

A common conception is that PCC is not suitable for certain healthcare settings, such as emergency healthcare, and is better suited for primary care settings. Unfortunately, PCC is often described by articulating what it is *not* and is then contrasted with a biomedical model focusing on organs and bodily functions. Kristensson Uggla^9^ discussed the two dominant knowledge cultures in healthcare: focusing on the patient as (a) an object (objective knowledge ‘from the outside’) and (b) a subject (subjective knowledge ‘from the inside’). Notably, PCC not only belongs to the subject side, despite its emphasis on the person, the narrative, and the patient as an expert but is also an intersection of the two. The patient as a person includes both the body as an object (function and organ) and as a lived experience (the patient's experiences). Thus, person‐centeredness and the biomedical model are not mutually exclusive and have overlapping goals.^4^ Understanding a patient's narrative and social context can provide clues to the aetiology of their disease and assist in correct diagnoses. Knowledge of a patient's values, circumstances and resources can indicate when certain treatment strategies are appropriate and whether patients are willing and/or able to follow them. PCC has been introduced in a variety of care contexts, including inpatient care, rehabilitation, outpatient care, school settings and primary care^10^ in patient groups with acute psychosis, acute coronary heart disease, hypertension, rheumatism, heart failure, hip fractures, long‐term pain and nursing home residents.^10^, ^11^

## 3 PCC IS THE SAME AS THE PATIENT DECIDING ON ALL ASPECTS OF THEIR HEALTHCARE

**Figure 1:**
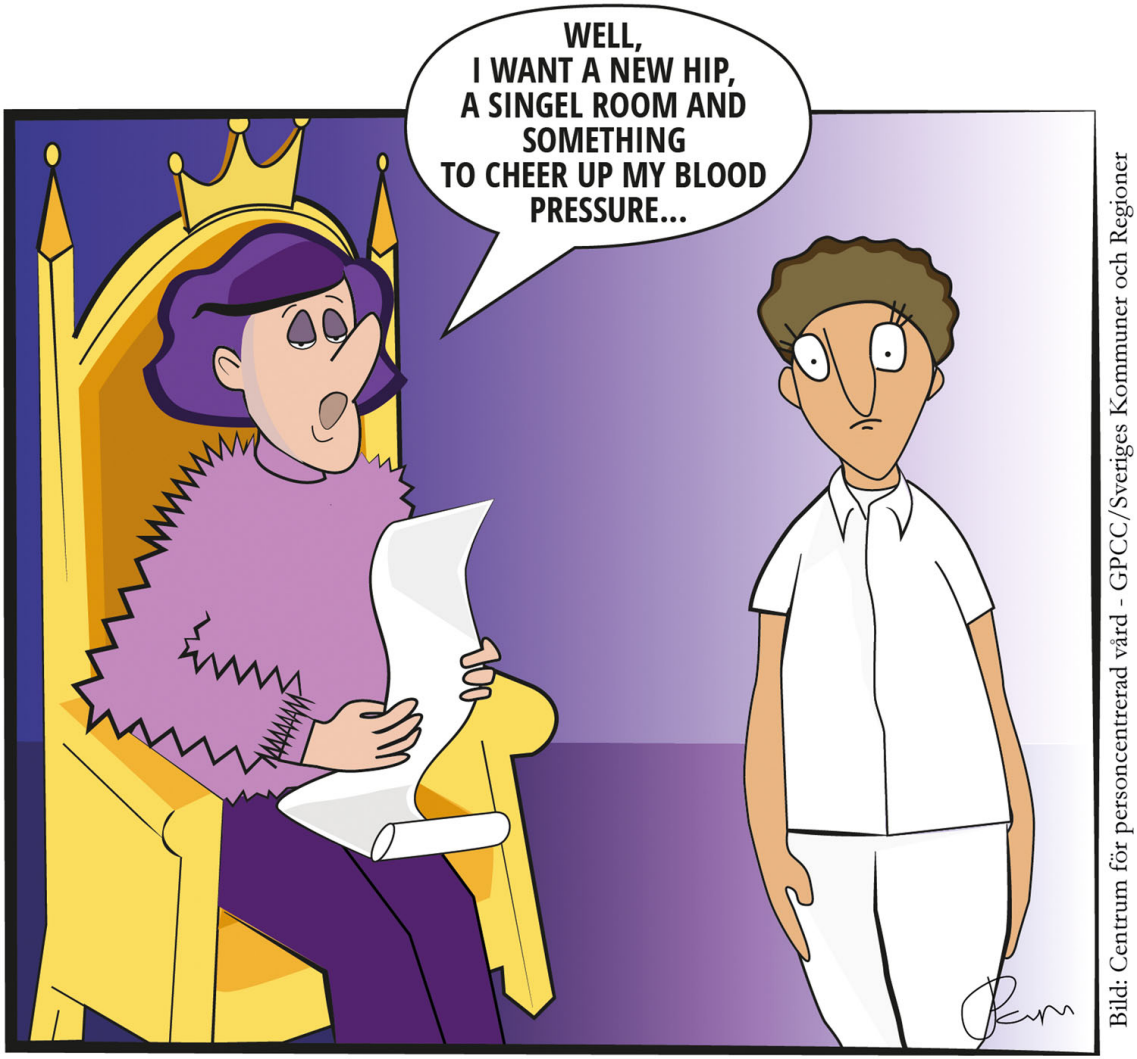
Illustration of the conception that person‐centred care is the same as the patient deciding on all aspects of their healthcare (created by Pamela Lindgren).

Issues of power and responsibility in the relationship between healthcare professionals and patients have been discussed for years, and changes in these factors have developed historically in parallel with other societal developments towards more egalitarian systems. There are three relationship types between patients and healthcare professionals: active–passive, guidance–collaboration and mutual participation.^12^ The first two are paternalistic and profession‐centred, while the latter is person‐centred. What does PCC imply in relation to the issues of power and responsibility? A review article found that *shared* responsibility was central to centeredness.^13^ The word ‘shared’ is important, with the authors stating that healthcare professionals, as experts on health and care, have a professional responsibility for their patients, while the patient's responsibility is based on ability and wishes.^13^ Professional responsibilities include, for example, ensuring that the prioritization principles that apply to healthcare are not replaced by the patient's wishes. Kristensson Uggla^9^,p.1 described it as a common notion that ‘a former all‐mighty doctor must now be replaced by an all‐mighty patient’. However, this is not the case since shared power and shared responsibility are the starting points for PCC. See Figure 1 for an illustration of this conception.

## 4 PCC IS TOO DEMANDING FOR MOST PATIENTS; THEY WANT HEALTHCARE PROFESSIONALS TO MAKE DECISIONS

This conception contains two aspects, with one being that patients do not want PCC but prefer to have healthcare professionals make decisions. However, when asked through citizen panels, people were positive about PCC.^14^ Therefore, do patients not want to participate, or are they not invited to do so? There is a history of healthcare systems that have not allowed patients to participate in their own care. Hence, the shift towards active patient participation is new, and patients may be unaccustomed to this role.^15^

The second aspect is a concern that PCC might favour already resourceful patients and that marginalized groups are thus disadvantaged by it. To date, such tendencies have not been demonstrated in the literature. Conversely, one study showed that PCC was most beneficial for those with the lowest educational levels.^16^ The focus on the patient narrative within PCC has also received criticism.^15^, ^17^ What does this mean for those with communication disorders or cognitive problems? However, there are several examples of how PCC can be implemented in different settings. For example, for elderly people with reduced cognitive abilities, family members were involved in their care, and information about the patient's habits, interests and wishes was collected through them.^18^ Having a patient as a partner does not mean that the patient should act as a medical expert. Patients should be experts on themselves, and in cooperation, if necessary, with the help of relatives, they can actively participate in their own care.^6^

## 5 WE ARE NO LONGER ALLOWED TO USE THE TERM ‘PATIENT’

The conception that PCC implies that healthcare professionals must say ‘person’ instead of ‘patient’ is not unusual. While there is no contradiction between the concepts, the terms are not interchangeable.^5^ Many of us are patients in certain situations and during certain periods, and at such times, the patient's role can be more prominent. In other situations, the role of parent or professional is more dominant, and the patient role is downplayed. When in a role, an individual is *something*, such as a parent, manager, doctor or nurse, while being a ‘person’ means being *someone*. Therefore, we are always a ‘person’ and sometimes a ‘patient’. In the healthcare context, using the word ‘patient may be appropriate’, even though the patient is always also a ‘person’. Notably, the term ‘patient’ is associated with a certain status in professional and legal steering documents, but it can also be associated with subordination and passivity.

## 6 WE DO NOT NEED TO IMPLEMENT PCC BECAUSE WE ALREADY WORK IN THAT MANNER

Many healthcare professionals believe that they already work in a person‐centred manner; therefore, they do not feel that they need more knowledge or should change their routines. This may be the case in some care units because many units have a stated ambition to work actively according to person‐centred ethics and practices. However, consistently working in a person‐centred manner in all situations at all times can be difficult because it requires awareness of each individual's actions, routines, and working methods. Therefore, it would be interesting to test this notion and ask how one can be sure that the unit works in a person‐centred manner. Does the unit work systematically with PCC (i.e., always and in all contexts)? Have they validated what patients think? Have they reached the optimal person‐centred way of working, or can it be further strengthened? The problem is that PCC does not have a clear definition and is used in different ways. However, even if there are difficulties in measuring whether PCC is implemented, there are tools that can be helpful.^19^ The standard from 2020 for patient participation can also be utilized (SS‐EN 17398: 2020).

## 7 WE HAVE NEITHER THE TIME NOR THE RESOURCES FOR PCC

Another common belief is that PCC takes more time and resources than current routines.

Regarding the implementation of PCC approaches, in one case bedside shift reports, nurses expressed apprehension about the potential time requirement of bedside handovers when compared to conventional methods.^20^, ^21^ The conception gives rise to many questions, such as: Is PCC more demanding compared to the introduction of other approaches? However, PCC does not have to lead to longer meetings than non‐PCC. On the contrary, studies indicate that PCC is cost‐effective.^22^, ^23^ PCC is rather about reconsidering the balance between the one who speaks and the one who listens so that the patient is given the opportunity to express their needs and resources.

## 8 CONCLUSION

Common conceptions of PCC are important to acknowledge and discuss as they can delay the implementation of PCC across healthcare settings and systems. These beliefs should be discussed and problematized to advance PCC knowledge. Discussions and reflections on PCC can help identify and clarify ambiguities and possible gaps between PCC as a theoretical construction and actual healthcare practices. Discussing and attempting to close the gap between ideal and plausible PCC practices might also reduce the ethical stress experienced by many healthcare professionals.

Some of the conceptions are discussed here, such as that PCC is the same as the patient making decisions about all aspects of their healthcare and that PCC is too demanding for most patients, which presupposes an either/or relationship (i.e., a dichotomy). We see it as a continuum that can be bridged by partnerships, shared responsibility, and a mutually agreed‐upon health plan.

## CONFLICT OF INTEREST STATEMENT

The authors declare no conflict of interest.

## Data Availability

Data sharing is not applicable to this article as no datasets were generated or analysed during the current study.
